# Supplementary material for: Hybrid Models and Biological Model Reduction with PyDSTool
Source: PLoS Comput Biol. 2012 Aug 9;8(8):e1002628. doi: 10.1371/journal.pcbi.1002628 (PMC3415397; doi:10.1371/journal.pcbi.1002628)
Supplement: Text S4 — Complete source code for the PyDSTool package (version 0.88.120504). Includes API documentation and help files linking to web pages. This file is identical to the current public release on Sourceforge.net. (ZIP) [file pcbi.1002628.s004.zip › PyDSTool/html/toc-PyDSTool.conf-module.html]

xml version="1.0" encoding="ascii"?


conf


# Module conf

---

## Variables

copyright  
 exclude\_patterns  
 extensions  
 html\_static\_path  
 html\_theme  
 htmlhelp\_basename  
 latex\_documents  
 man\_pages  
 master\_doc  
 project  
 pygments\_style  
 release  
 source\_suffix  
 templates\_path  
 version  

---

[hide private]
